# Temperate-Tropical Variation in Breeding Synchrony and Extra-Pair Paternity Among New World *Tachycineta* Swallows

**DOI:** 10.1038/s41598-019-48980-x

**Published:** 2019-09-03

**Authors:** Valentina Ferretti, Viviana Massoni, Marcela Liljesthröm, Mariela V. Lacoretz, David W. Winkler

**Affiliations:** 1 Fuller Evolutionary Biology Program, Cornell Lab of Ornithology, 159 Sapsucker Woods Rd, Ithaca, NY 14850 USA; 2 Department of Ecology and Evolutionary Biology, Corson Hall, Cornell University, Ithaca, NY 14853-2701 USA; 3 Present Address: Instituto de Ecología, Genética y Evolución de Buenos Aires-CONICET, Facultad de Ciencias Exactas y Naturales, Universidad de Buenos Aires, Pabellón 2 - Ciudad Universitaria, Av. Int. Güiraldes 2160, C1428EGA Buenos Aires, Argentina; 4 Centro Austral de Investigaciones Científicas, CONICET, Bernardo Houssay 200, V9410BFD Ushuaia, Tierra del Fuego Argentina; 5 Departamento de Ecología, Genética y Evolución, Facultad de Ciencias Exactas y Naturales, Universidad de Buenos Aires, Pabellón 2 - Ciudad Universitaria, Av. Int. Güiraldes 2160, C1428EGA Buenos Aires, Argentina

**Keywords:** Evolutionary ecology, Sexual selection

## Abstract

Extra-pair paternity rates vary markedly across avian taxa, but patterns of variation in this trait have been obscured by a paucity of data on closely related species, especially those spanning broad environmental gradients. Here we compare variation in extra-pair paternity rates among five species in the widespread swallow genus *Tachycineta*. Rates of extra-pair paternity vary widely in this group, ranging from 13 to 87% of nests having extra-pair young. The inter-specific variation in extra-pair paternity within this small group of closely related swallows has a range equivalent to that found among all Hirundinidae and is close to the range of variation across all birds. Despite theory that predicts extra-pair paternity rates to be explained by latitudinal variation in breeding synchrony our results show that extra-pair paternity rates in this genus do not closely track a latitudinal gradient, as predicted by studies of other life-history traits, and are not explained by differences in breeding synchrony as previously suggested. The genetic mating systems of birds, described by the rates of extra-pair paternity, are connected to all other life-history traits through a complex network of trade-offs with organismal (phylogenetic) and ecological (environmental) factors. Disentangling each of these interactions to understand latitudinal patterns in any given life-history trait remains a daunting task.

## Introduction

Broad geographic patterns have long intrigued researchers interested in the evolutionary and ecological determinants of variation in life-history traits in birds^1^. Early contributors to the development of avian life-history theory documented latitudinal clines in life-history characters and provided a variety of ecological hypotheses to explain this variation, suggesting that diversification in life-history strategies is related to *current* ecological factors that co-vary with environmental heterogeneity^2–4^. Alternatively, comparative studies on birds found that the variation observed today in these traits probably reflects *ancient* ecological selective factors that played a key role in the radiation of this group, with more than 50% of the inter-specific variation being explained by differences at the taxonomic level of Family and Order^5–7^. Yet, we still do not have a clear understanding of the diversification of avian life-history traits. On one hand, substantial variation exists below the level of Family (see for example variation in extra-pair paternity, EPP, rates in^8,9^) suggesting that there are current factors influencing the evolution of life-history traits and that we still need to fathom the interaction between these and the evolutionary history of the taxa under consideration. In fact, in the case of EPP rates, one limitation of comparative studies of variation in this trait is that there are very few closely related groups of species that can be analysed, and there is likewise a marked geographic bias, with most species sampled from populations in Europe and/or North America (see appendix in^6^), and this is true for many other traits as well (e.g.^5^). On the other hand, ecological hypotheses have been proven hard to test in the wild. For example, one of the ecological hypotheses proposed to explain inter-specific variation in EPP rates in birds is based on geographic variation in breeding synchrony^10^. Under this hypothesis, high breeding synchrony leads to high EPP rates because females will be better able to compare the quality of potential mating partners when breeding is synchronous in the population, facilitating their extra-pair mating decisions. Therefore, current ecological factors affecting female synchrony in the breeding populations will have an indirect effect on the females’ mating decisions and the rates of EPP. An association between breeding synchrony and EPP might also lead to broad-scale geographic variation in EPP rates, with rates generally lower for species breeding in the tropics than for those breeding at higher latitudes^10,11^. However, the predictions made by this hypothesis have been hard to test in most traditionally territorial birds: the many studies that have examined the correlation between breeding synchrony and paternity rates among individuals in the same breeding population and season have yielded conflicting results (reviewed in^12,13^). This is in part because within-season studies are weak tests for a positive relationship between synchrony and EPP, as females might be evaluating males in periods of high breeding synchrony and basing their mating decisions on this prior evaluation^14,15^. In fact, the best test for this hypothesis might be to compare different populations of the same species breeding at different latitudes having different degrees of breeding synchrony^11,14^. Studies including many populations and species are expensive and logistically complicated to carry out, and thus very rare, hindering the evolutionary and ecological interpretation of variation in life-histories. While there have been several studies addressing the relevance of this hypothesis in explaining geographic variation in EPP rates (e.g.^6,7,14,16,17^) we argue that the restricted geographic distribution of the species used in the analyses limits the interpretation of the results.

The focus of our study is to analyse geographic variation in EPP rates in a group of closely related birds and test the breeding synchrony hypothesis. We explore here the genetic mating system of five species in the swallow genus *Tachycineta* (Tree swallow *T. bicolor*, Violet-green swallow *T. thalassina*, Mangrove swallow *T. albilinea*, White-rumped swallow *T. leucorrhoa*, and Chilean swallow *T. meyeni*) that span a wide breeding distribution in North, Central and South America. Previous studies on four species in this genus have shown that *Tachycineta* swallows have high variation in their rates of EPP, ranging from 13 to 87% of nests having extra-pair young^18–21^. In this study, we more fully document inter-specific variation in EPP among *Tachycineta* swallows by (i) characterizing for the first time the genetic mating system of Violet-green swallows; (ii) describing the rate of EPP in additional populations of Mangrove and White-rumped swallows; (iii) examining the geographic pattern of variation in EPP rates and testing the latitudinal variation in this trait; and (iv) testing the breeding synchrony hypothesis along a latitudinal gradient. This analysis of variation among closely related *Tachycineta* species allows us to simultaneously evaluate diversification in EPP rates from a historical and a contemporary ecological standpoint. To our knowledge this is the first and most comprehensive study of genetic mating system of several members in a taxon with a widespread New World distribution, spanning Northern, tropical and Southern latitudes.

## Results

### Characterization of the genetic mating system of tachycineta species

**Figure 1:**
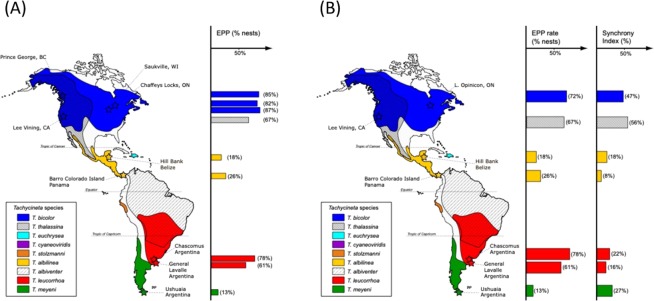
Map of the ranges of the nine species of *Tachycineta* swallows. (**A**) Bars on the right represent per cent of nests with extra-pair young for the colonies sampled, including the ones reported for the first time in this study and those taken from the literature. (**B**) Bars on the right represent per cent of nests with extra-pair young for the colonies sampled and the per cent of breeding synchrony in the colony for the years sampled (note that EPP rates for Tree swallows differ in this respect between the two maps, this is because the study used for the reported synchrony showed a somewhat lower EPP rate for other years studied). Bars are coded by species (see colour codes) and are located at the approximate latitudes of the populations sampled.

Here we report for the first time rates of EPP for the Violet-green swallow. This species had very high rates of EPP with 67% of nests with at least one extra-pair young, and 56% extra-pair young in the population. Violet-green swallows breeding in California had between 1 and 4 extra-pair young in their nests, sometimes accounting for all the offspring in a brood. In addition, Mangrove swallows breeding in Belize had between 1 and 2 extra-pair young in their nests in 18% of nests in the population, and 7% of the offspring produced were a result of extra-pair copulations. Extra-pair offspring never accounted for all nestlings in a brood at this site. Finally, 61% of nests of White-rumped swallows breeding in General Lavalle, Buenos Aires province, had at least one extra-pair young, and 35% of offspring in the population were a result of extra-pair behaviour, with 2–3 extra-pair nestlings per brood (Table 1, Fig. 1A), sometimes accounting for all the offspring in a brood.

**Table 1:** Newly measured extra-pair paternity rates for three species of *Tachycineta* swallows.

Species	Total number of nests	Total number of nestlings	Nests with extra-pair offspring	Extra-pair offspring
VGSW	27	78	18 (67%)	44 (56%)
MANS	22	95	4 (18%)	7 (7%)
WRSW	13	51	8 (61%)	18 (35%)

VGSW = Violet-green swallow, MANS = Mangrove swallow, WRSW = White-rumped swallow.

### Geographic variation in breeding synchrony and EPP rates

**Figure 2:**
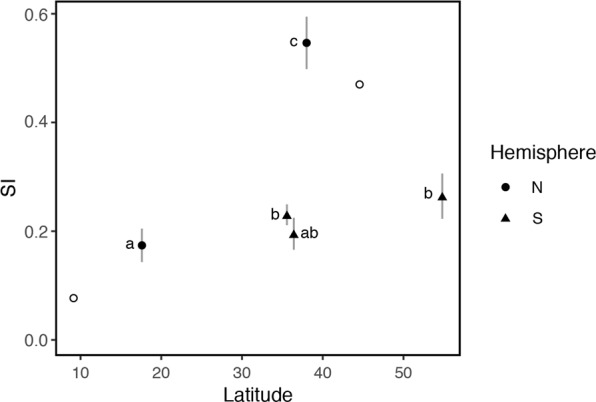
Latitudinal variation in population synchrony index (SI, calculated following^49^). X axis: absolute latitude; Y axis: SI. Open symbols represent population means taken from previously published studies; filled symbols represent the population means calculated by the model for the data used in this study (n = 406, from 5 different populations). Lines represent the 95% confidence intervals for the different SIs estimated. Populations from the North hemisphere are represented by circles, populations from the South hemisphere are represented by triangles. Symbols with different letters have statistically different SI values.

There is substantial geographic variation in EPP and breeding synchrony (represented here by a synchrony index, SI) for all *Tachycineta* populations sampled to date (Fig. 1). For SI we worked with breeding information for 406 females from 5 different populations (Table 2). In the final model, we found that SI presented differences among the various latitudes considered, but this variation did not follow a latitudinal gradient (Fig. 2, Supplementary Tables S2 and S3). Birds breeding at similar intermediate latitudes (i.e., 30°–40°) had significantly different SI. At the same time, while birds in the North showed an increase in SI with latitude (filled circles, Fig. 2, Supplementary Table S3), birds in the South did not show differences in SI with increasing latitude (filled triangles, Fig. 2, Supplementary Table S3).

**Table 2:** Final sample sizes of nests/females used for the glm analysis with complete individual female information for the breeding synchrony (SI) model.

Total number sampled by species:		Total number sampled by location:	
WRSW	260	Chascomús, Argentina	192
		General Lavalle, Argentina	68
MANS	55	Hill Bank, Belize	55
VGSW	46	Lee Vining, California	46
CHSW	45	Ushuaia, Argentina	45

Note that we did not use Tree swallows on this analysis, in spite of the fact that there are many papers on their genetic parentage, because we did not have access to individual female information from the papers used as reference. VGSW = Violet-green swallow, MANS = Mangrove swallow, WRSW = White-rumped swallow, CHSW = Chilean swallow.

**Figure 3:**
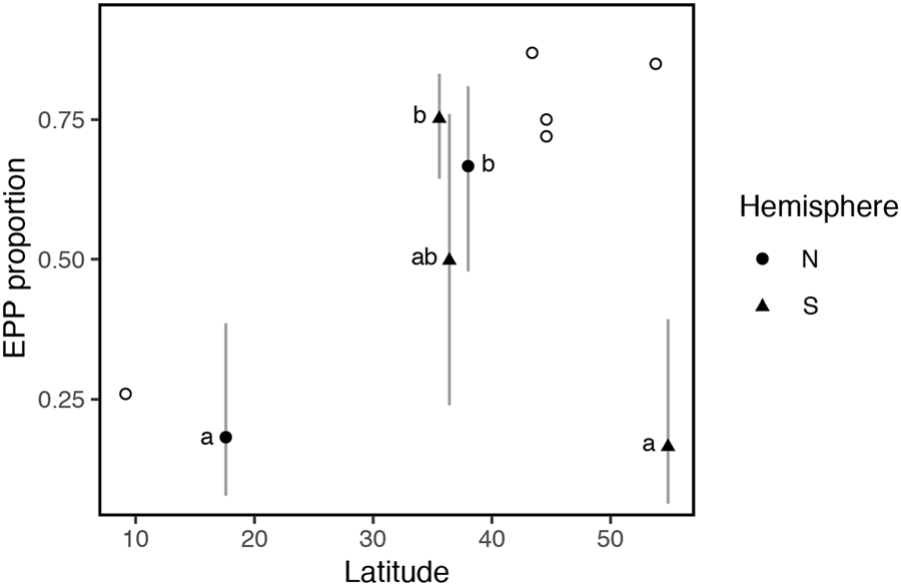
Latitudinal variation in the proportion of nests with extra-pair offspring in each population. X axis: absolute latitude; Y axis: proportion of females in the population with at least one extra-pair offspring in their nest (EPP proportion). Open symbols represent population proportions taken from previously published studies; filled symbols represent population proportions calculated by the model for the data used in this study (154 nests from 5 locations). Lines correspond to the 95% confidence intervals for each proportion calculated following Wilson’s method used for binomial variables with small sample sizes^58^. Populations from the North hemisphere are represented by circles, populations from the South hemisphere are represented by triangles. Symbols with different letters have statistically different proportion values.

For EPP we worked with 154 nests from 5 different latitudes (Table 3). In the final model, latitude was also important (Fig. 3, Supplementary Table S2), but the effect of SI as an explanatory variable was not statistically significant. When we compared populations by pairs, we found that for birds breeding in the North there was an increase in the rate of EPP with latitude (filled circles, Fig. 3, Supplementary Table S4), but there seemed to be a decrease in this variable with latitude in the South (filled triangles, Fig. 3, Supplementary Table S4). Moreover, the populations located at 17°36′N and 54°44′S had similar rates of EPP despite the enormous variation in latitude.

**Table 3:** Final sample sizes of nests/females used for the glm analysis with complete individual female information for the extra-pair paternity (EPP) model.

Total number sampled by species:		Total number sampled by location:	
WRSW	87	Chascomús, Argentina	77
		General Lavalle, Argentina	10
MANS	22	Hill Bank, Belize	22
VGSW	27	Lee Vining, California	27
CHSW	18	Ushuaia, Argentina	18

Note that we did not use Tree swallows on this analysis, in spite of the fact that there are many papers on their genetic parentage, because we did not have access to individual female information from the papers used as reference. VGSW = Violet-green swallow, MANS = Mangrove swallow, WRSW = White-rumped swallow, CHSW = Chilean swallow.

Figures 2 and 3 correspond to the data used in our analyses; however, we added to the plot the values of the averages taken from the literature, but not included in our tests (shown in open circles).

## Discussion

Understanding patterns of variation in life-history traits (e.g., EPP) requires looking at within-population variation as well as variation between species and populations. Most studies on avian EPP rates to date have been restricted to single populations and single species within most genera, limiting our understanding of how evolutionary and environmental changes can affect genetic mating systems^6,22^. The North temperate species Tree swallow has long been the focus of research of behavioural ecologists, in part because of its extremely high rates of EPP^23^ (Table 4). The contrasting finding of low rates of EPP in the tropical Mangrove swallow and the southernmost species, Chilean swallow (Table 4), makes a compelling case for studying variation in EPP rates between closely related species in this genus, providing us with a unique dataset for studies of variation in paternity.

**Table 4:** Rates of extra-pair paternity for four *Tachycineta* species taken from the literature, with the latitudes of all populations sampled.

Species	EPP nests	EPY	Latitude of colony	Reference
TRES	82%	51%	44°34′N	(a)^18,59–61^
	87%	49%	43°23′N	(b)^62–64^
	85%	35%	53°N	^65^
VGSW			37°58′N	**This study**
MANS	26%	15%	9°10′N	^21^
			17°36′N	**This study**
WRSW	78%	55%	35°34′S	^19^
			36°25′S	**This study**
CHSW	13.5%	6.8%	54°44′S	^20^

EPP nests: % of nests with extra-pair young. EPY: % of extra-pair nestlings in the population. TRES = Tree swallow, VGSW = Violet-green swallow, MANS = Mangrove swallow, WRSW = White-rumped swallow, CHSW = Chilean swallow.

(a) We calculated the averages for this population using the results reported on these four studies; (b) We calculated the averages for this population using the results reported on these three studies.

### Characterization of the genetic mating system of Violet-green swallows

We sampled a population of Violet-green swallows breeding in Lee Vining, California. We found very high rates of EPP in this population (67% of nests and 56% of nestlings), comparable to those of White-rumped swallows breeding at a similar latitude in the South (Table 1). Despite the many studies on Tree swallows in North America, it is surprising that their congener, the Violet-green swallow, had been largely neglected in studies of mating systems, especially since both species overlap in part of their range^24,25^. This is, to our knowledge, the first study to describe the genetic mating system of this species. We used this information on the analyses of latitudinal variation in EPP and SI.

### Geographic variation in EPP rates and a test of the breeding synchrony hypothesis

We found considerable geographic variation in EPP rates among different populations of the same species of *Tachycineta* swallows (e.g., 61 to 78% nests with extra-pair young in WRSW, Tables 1 and 4), as well as between closely related species breeding at different sites (e.g., 13 to 78% of nests with extra-pair young for the sister species Chilean swallow and White-rumped swallows, respectively, Table 4, Fig. 1A and Supplementary Fig. S1). Geographic differences in EPP have been predicted to follow a latitudinal pattern of temperate/tropical variation related to latitudinal variation in SI^10,11^. Our results show, however, that differences in EPP are not explained by differences in SI: we did not detect an effect of SI on EPP. Moreover, the breeding synchrony hypothesis proposed to explain latitudinal variation in EPP requires synchrony to follow a latitudinal gradient increasing towards higher latitudes, and EPP to follow this same positive association with increasing latitude^10,11,26^. Yet, we did not detect a strong effect of latitude on SI across hemispheres—only in Northern hemisphere populations SI increases with latitude in our data (Figs 1A and 2)—and we detected a contrasting pattern of latitudinal variation in EPP between hemispheres (Figs 1 and 3), with species in the Southern hemisphere having a negative relationship, and species in the North having a positive relationship with latitude. The addition of closely related species breeding at different sites and, in particular, species breeding in the Southern hemisphere uncovered a different pattern of variation from the one proposed by Stutchbury^14,15^ and Stutchbury and Morton^10,11^ in general, and by Moore *et al*.^21^ in species of this same genus. A possible explanation for our results is that SI might not fully explain variation in EPP. While SI might be related to mating behaviour, other ecological variables can play a preponderant role in shaping mating systems. For example, SI is linked to seasonality, but seasonality responds to a combination of weather patterns, dependent on geography (i.e. altitude, continentality and/or distance to the ocean); and seasonality might affect adult survival, which in turn, will co-vary with latitude most of the time, although not always. In fact, variation in adult survival has been proposed as an alternative explanation for variation in genetic mating systems, with low adult survival related to high EPP rates^27^—under low adult survival, tolerance of EPP should increase because of the high chance of not breeding again—and EPP might follow the pattern of variation in this trait. In particular, in this genus, Chilean swallows breeding at 54°S have the highest apparent adult survival rates in the group and lowest EPP rates, while Violet-green swallows, White-rumped swallows and Tree swallows have the lowest survival rates (DWW unpub data, and^28–30^), and high EPP rates (Table 4), which could explain variation in EPP rates in *Tachycineta*. Another possible explanation for the geographic variation in EPP, is that it is not breeding synchrony *per se* what drives rates of EPP, but rather the length of the breeding season and migratory behaviour—longer seasons may give females more time to choose mates and even breed multiple times, favoring selection for faithful males and females, given the potential benefits of staying with the same partner, while migration coupled with shorter seasons can lead to haste in mate choice followed by extra-pair behaviour^31,32^. We did not find, however, a correlation between rates of EPP and length of the breeding season in the populations studied (Supplementary Table S5). In fact, while resident Mangrove swallows tend to breed with the same partners in consecutive breeding seasons, with a small proportion of pairs being double brooded (DWW and VF unpub data), migratory Chilean swallows had the lowest rates of EPP with a shorter breeding season.

Our findings might be strongly driven by data from the Chilean swallow population sampled and/or by the scarcity of sites at intermediate latitudes in both hemispheres (see Fig. 1). The population of Chilean swallows breeding in Tierra del Fuego may be an outlier to a general latitudinal trend^20^. Other life-history traits in this population (i.e., unexpectedly small clutch size, high adult survival^29,33^) do not fit the pattern of geographic variation described in multi-species studies^34^ and many aspects of the breeding biology of this population may be responses to the distinctively extreme climate of the Fuegian breeding site. One of the challenges of the latitudinal hypothesis is that it does not specify exactly which environmental features act proximally to cause a latitudinal pattern of variation. As mentioned above, the other important caveat is the lack of studies from sites at intermediate latitudes in both hemispheres, which might respond mainly to the concentration of researchers at other latitudes. Further field work, concentrated on sites with extreme sets of environmental variables as well as those at intermediate latitudes, could help elucidate which of the many factors that change with latitude are likely to be most important. One consequence of such further work might be that Fuegian Chilean swallows will come to be seen as only one of many exceptions to a latitudinal trend, or that Chilean swallows will serve as indicators of a relationship between EPP and some presently unappreciated aspect of their biology. Indeed, it may be that studies at a few more sites will be sufficient to erase any suggestion of a general latitudinal trend. We currently do not have enough information and sites sampled to tease apart these two alternatives.

Life-history traits form a complex interconnected network of relationships, and the resulting strategies we observe and measure in nature are no more than the adaptive outcomes of these complex trade-offs among traits responding to geographically different selective pressures (see Figs 2 and 3 in^35^). Therefore, the isolation of one trait or one ecological variable cannot provide the full insight we seek in the understanding of avian life-history variation, including mating systems. This has been clearly stated in Brouwer *et al*.’s study^8^ where several predictors explained levels of EPP in the family Maluridae, a family of five genera restricted to Australia and Papua New Guinea^36^. Our study, comprising members of the same genus in North, Central and South America, provides additional comparative evidence that breeding synchrony alone cannot explain the pattern of variation in EPP observed in nature.

### A note on historical EPP variation in the genus *Tachycineta*

We found extreme variation in rates of EPP in *Tachycineta* (13–87% broods with extra-pair young, Table 4, Fig. 1). The extensive EPP variation found within this genus encompasses the range of variation found in the entire Family Hirundinidae^9^, and close to the variation found across all of Aves (0–95%^6^). It is clear from previous comparative studies that variation in avian genetic mating patterns can have a phylogenetic component^6,7^. However, the substantial variation in this trait, found among the tips of the phylogenetic tree, among closely related birds (Supplementary Fig. S1, see also^9^) reminds us that we do not have yet a full understanding of when or how differentiation in EPP rates has occurred. A notable example of this variation is found between the sister taxa White-rumped swallows and Chilean swallows, with 78% and 13% of nests with extra-pair young respectively (Fig. 1, Table 4). An integrated view of the partitioning of the variance in avian mating systems across levels of relationship must await a more thorough sampling at shallow phylogenetic levels, especially among closely related species. For example, work by Kingma *et al*.^37^ found very low levels of EPP in the Purple-crowned fairy-wren (5.8% of the broods containing extra-pair young), a member of the genus *Malurus*, otherwise known for its high levels of promiscuity. Interestingly, this substantial exception from the genetic mating system in *Malurus* was not associated with changes in other life-history traits hypothesized to drive interspecific variation in EPP. These results, like those in the current study of *Tachycineta*, indicate that extra-pair mating systems, though subject to a phylogenetic influence^6,12^, can be quite labile evolutionarily.

## Conclusion

Comparative studies of biological traits have informed our understanding of the timing and factors involved in the diversification of life-history strategies. At the same time, field studies have provided great insight into the importance of ecological variables as drivers of variation in life-history traits. However, an integrative explanation of variation in EPP still remains elusive. The large variation of EPP rates within the pan-American *Tachycineta* clade, and between years in the same population of at least some species (e.g.^18^), indicate that neither phylogenetic history nor geography alone can explain variation in genetic mating systems. More comparative studies of closely related species that span strong environmental gradients are needed to increase our understanding of the broad patterns of variation in extra-pair mating systems and life-history traits in general. The idiosyncrasies of geographical ecology can shape life-history strategies in ways that may be too complex to disentangle with standard methods of analysis and hypotheses that address only one of many potential causes of avian life-history variation. In addition we need to be aware of, and guard against, North-temperate biases that pervade our world-view and the theory that it produces^38^. We need hypotheses that spring from the fact that most diversification we see today in Aves is located in the tropics and Southern hemisphere^39–41^. A continued view that the patterns observed in Northern species are the “norm” and that tropical and Southern species are “different” will unavoidably obscure our understanding of evolutionary ecology.

## Methods

### Colonies and species

The nine species in the New World genus *Tachycineta* are distributed throughout the Americas and the Caribbean^24,25^. In this study we included five of the species with continental distributions, with samples spanning a range of latitudes from 53°N (British Columbia) to 54°S (Tierra del Fuego). We used previously reported paternity and latitudinal data for three populations of Tree swallows in the North, one population of Mangrove swallows in the tropics, one population of White-rumped swallows and one population of Chilean swallows in the South (see Table 4 for citations). We generated new information for one previously uncharacterized species—Violet-green swallows from the Western United States—an additional population of Mangrove swallows in Central America, and an additional population of White-rumped swallows in Buenos Aires. These populations were studied from 2003–2004 for White-rumped swallows, 2003 for Mangrove swallows, and 2008–2009 for Violet-green swallows. Table 4 provides details on the locations of the breeding colonies used in this analysis.

### Standardized field protocols for sampling

Details on sampling protocols and nest-box spacing for the previously reported populations can be found in the references in Table 4. For other colonies used in our study, nest-boxes were spaced 20–35 m apart and checked every other day for the length of the breeding season. We captured both adult breeders inside the nest boxes using box traps for every nesting attempt. All captured individuals were measured, bled, and banded with aluminium bands. When nestlings were 7–9 days old, we banded them with aluminium bands and took a blood sample from each. We took 20–70 µl of blood from both adults and nestlings, collected using a heparinized capillary tube via brachial venipuncture, and then stored whole blood in lysis buffer^42^. When nestlings were found dead in the nest before they were banded and bled, we collected a sample from their pectoral muscle and stored it in 96% ethanol.

### Genetic paternity analyses for violet-green, mangrove and white-rumped swallows

We extracted DNA from blood and muscle samples using DNA purification kits by Eppendorf and Qiagen. Extracted DNA was diluted 1:10 in ultrapurified H_2_O and then amplified at a panel of highly polymorphic microsatellite loci^43–46^, which differed somewhat across species (see the Supplementary Table S1 for combination of loci used for each species, primer concentrations, details on polymerase chain reaction (PCR) concentrations, conditions and cycling profiles). PCR products were then genotyped on an ABI PRISM 3100 Genetic Analyzer (Applied Biosystems), and the sizes of the microsatellite alleles estimated using GeneScan-500 LIZ size standard (Applied Biosystems) and the software GeneMapper (v3.7 Applied Biosystems).

We used the program Cervus 3.0^47,48^ to generate allele frequencies and population genetic parameters, and assess paternity for the populations studied. The combined exclusion probability for all loci used for each species was >0.9999. Species-specific details on the maximum likelihood assessment of paternity can be found in the Supplementary Methods.

We first compared the nestlings’ genotypes with the genotype of the adult female attending their nest (i.e., the putative mother). As expected, all nestlings shared at least one allele at each of the amplified loci with their putative mother. The nestlings’ genotypes were then compared to those of their putative father. We considered nestlings to be extra-pair young when they mismatched the social father’s genotype at two or more loci.

### Synchrony measures

We calculated a breeding synchrony index (or “SI”) using Kempenaers’^49^ formula, which represents the proportion of fertile females in the population that overlapped with each female’s fertile period. For the calculation we used the number of fertile days for each female defined as six days prior to the laying of the first egg^50^ up to the day the penultimate egg was laid. Average SIs varied considerably across populations (Table 5), and we analysed each female’s SI using generalized linear models as described below.

**Table 5:** Female breeding synchrony and latitude for the seven populations of *Tachycineta* species used in this study.

Species	SI (%)	N (nests)	Latitude of colony sampled	References
TRES	47	57	44°34′N	^21^
VGSW	55.64	46	37°58′N	This study
MANS	7.7	48	9°10′N	^21^
	17.92	55	17°36′N	This study
WRSW	22.58	192	35°34′S	This study
	16.07	68	36°25′S	This study
CHSW	27.28	45	54°44′S	This study

SI: synchrony index calculated following^49^; N: number of nests sampled. TRES = Tree swallow, VGSW = Violet-green swallow, MANS = Mangrove swallow, WRSW = White-rumped swallow, CHSW = Chilean swallow.

### Data presentation and data analysis

We used population-wide rates of EPP and average SI to map these variables relative to latitude, but the statistical analyses of latitudinal variation in SI and EPP were limited to those populations for which we had individual female information. All statistical analyses were done in R, version 3.5.1^51^.

To assess variation in SI among the different latitudes we used a generalized linear model (GLM^52^), and considered latitude a categorical variable in this analysis. We assumed a beta binomial distribution because we obtained overdispersion when modelling with a binomial error distribution^53,54^. The response variable was modelled with a logit link function, using the R package and function glmmTMB^55^.

We also assessed variation in EPP, considered a dichotomous variable in our analyses (i.e., females that had extra-pair young in their nests, and females that did not have any extra-pair young in their nests), with respect to the explanatory variables latitude and SI. We used a GLM, with a binomial error distribution and a logit link function^52^, and used the glm function of the stats package in R^51^.

In both analyses we followed a hypothesis testing approach for model selection, using the drop1 function of the stats package^51^, which drops one explanatory variable at a time, and each time it applies an analysis of deviance test^56^. In the two models we conducted *a posteriori* Tukey’s all-pairs comparisons to look for differences in the response variables between the different latitudes. We used the package multcomp with the glht function for this^57^.

### Ethical approval and informed consent

All the procedures and experiments of our study comply with the current laws of the countries and provinces where the study was performed, and were performed in accordance with relevant guidelines and regulations of the different licencing committees involved. VF and DWW worked while covered by an approved animal welfare protocol (#2001-0051) at Cornell University to DWW, and by an approved protocol and permit issued by the Government of Belize’s Forestry Department dependent of the Ministry of Agriculture, Fisheries, Forestry, the Environment, and Sustainable Development, ML worked while covered by an approved permit from the Secretaría de Ciencia y Técnica (Science and Technology Governmental Office) of the Province of Tierra del Fuego, Argentina, and VM worked while covered by an approved permit from the Dirección de Fauna (Fauna Governmental Office), Province of Buenos Aires, Argentina.

## Supplementary information

Supplementary figures, tables and methods

## Data Availability

All data generated or analysed during this study are included in this published article (and its Supplementary Information files).
