# Supplementary material for: Temperate-Tropical Variation in Breeding Synchrony and Extra-Pair Paternity Among New World Tachycineta Swallows
Source: Sci Rep. 2019 Sep 3;9:12713. doi: 10.1038/s41598-019-48980-x (PMC6722081; doi:10.1038/s41598-019-48980-x)
Supplement: Supplementary file 1 — Supplementary figures, tables and methods [file 41598_2019_48980_MOESM1_ESM.docx]

temperate-tropical variation in breeding synchrony and extra-pair paternity among new world *Tachycineta* swallows

**Valentina Ferretti*, Viviana Massoni, Marcela Liljesthröm, Mariela V. Lacoretz and David W. Winkler**

* questions regarding these data should be addressed to vferretti@ege.fcen.uba.ar

**Supplementary information**

**Supplementary Figure S1** Phylogenetic relationships and rates of extra-pair paternity across *Tachycineta*. Grey boxes represent the per cent of nests with extra-pair young for the five species that have been surveyed to date. Phylogenetic relationships taken from Whittigham et al. (2002). EPN: nests with at least one extra-pair nestling.

**Supplementary Table S1** Microsatellite primers and conditions used for genotyping *T. albilinea*, *T. meyeni* and *T. thalassina*. N: number of unrelated individuals genotyped, Na: number of alleles, Ho: observed heterozygosity, He: expected heterozygosity, Ann Temp: annealing temperature. References: (a) Makarewich et al. 2009; (b) Richardson et al. 2000; (c) Crossman 1996; (d) Dawson et al. 2000; (e) Stenzler 2001

| Species | Locus | N | Na | Ho | He | Ann Temp | Primer  concentration | Ref. |
| --- | --- | --- | --- | --- | --- | --- | --- | --- |
| *T. meyeni* | Tabi1  Tabi4  Tal6  Tal8  Tle17  Tle19  Tle21  Tle4  Tle8  Tle11 | 56  56  56  56  56  56  56  56  56  56 | 8  8  4  35  6  4  4  25  8  18 | 0.857  0.768  0.268  1.000  0.500  0.446  0.250  0.946  0.714  0.804 | 0.783  0.731  0.254  0.964  0.502  0.411  0.229  0.947  0.690  0.745 | 56  56  58  58  56  56  58  56  56  60 | 1.2pM  4.8pM  1.6pM  3.6pM  2.4pM  1pM  1.2pM  2.4pM  1.2pM  1.2pM | (a)  (a)  (a)  (a)  (a)  (a)  (a)  (a)  (a)  (a) |
| *T. albilinea* | Tabi1  Tabi8  Tle19  Tle4  Tle11  Tal7  Tal8  Tle14 | 48  48  48  48  60  48  48  48 | 8  9  6  15  21  13  11  10 | 0.563  0.604  0.771  0.792  0.708  0.771  0.771  0.729 | 0.561  0.805  0.675  0.853  0.939  0.808  0.744  0.660 | 56  56  56  56  60  58  58  60 | 1pM  3.6pM  1pM  2.4pM  2pM  2.4pM  2pM  3pM | (a)  (a)  (a)  (a)  (a)  (a)  (a)  (a) |
| *T. thalassina* | Tabi1  Tabi8  Tal11  Tal6  Tal7  Tle16  Tle19  Ase29  MP3-31  MP5-29  Pca3  Tbi104  Tbi81 | 20  20  20  20  20  20  20  20  20  20  20  20  20 | 8  7  5  5  23  7  11  4  12  3  4  7  3 | 0.842  0.789  0.357  0.474  0.929  0.684  0.842  0.600  0.800  0.250  0.300  0.750  0.150 | 0.878  0.748  0.324  0.649  0.984  0.698  0.871  0.572  0.908  0.229  0.276  0.714  0.309 | 56  56  58  58  58  56  56  56  56  60  56  60  60 | 1.2pM  3.6pM  1.6pM  3pM  1.2pM  1.2pM  1.2pM  1.2pM  1.2pM  1.2pM  2.4pM  1.4pM  1.4pM | (a)  (a)  (a)  (a)  (a)  (a)  (a)  (b)  (c)  (c)  (d)  (e)  (e) |

**Supplementary Table S2** Results of GLM with beta binomial (SI) and binomial (EPP) error distributions for final models, assessing the effects of the different latitudes (Lat) on SI and EPP (see Methods section for details on the statistical analysis).

|  | SI final model | | | |  | EPP final model | | | |
| --- | --- | --- | --- | --- | --- | --- | --- | --- | --- |
|  | **Coefficient** | **SE** | ***z*** | ***P*** |  | Coefficient | SE | *z* | *P* |
| Intercept | -1.57 | 0.11 | -14.37 | <0.0001 |  | -1.50 | 0.55 | -2.72 | <0.01 |
| LatN_38 | 1.76 | 0.15 | 11.89 | <0.0001 |  | 2.20 | 0.69 | 3.20 | <0.01 |
| LatS_35.57 | 0.36 | 0.12 | 3.00 | <0.005 |  | 2.62 | 0.61 | 4.28 | <0.0001 |
| LatS_36.42 | 0.14 | 0.14 | 1.01 | 0.31 |  | 1.50 | 0.84 | 1.79 | 0.07 |
| LatS_54.81 | 0.54 | 0.15 | 3.50 | <0.0005 |  | -0.11 | 0.84 | -0.12 | 0.90 |

**Supplementary Table S3** Tukey’s contrasts for SI at the different latitudes considered. Each latitude is represented by the term LatN or S, corresponding to the hemisphere, followed by the absolute latitudinal value.

Estimate Std.Err z value Pr(>|z|)

LatN_38 - LatN_17.6 1.7601 0.1481 11.886 < 0.001

LatS_35.57 - LatN_17.6 0.3624 0.1207 3.002 0.02

LatS_36.42 - LatN_17.6 0.1456 0.1434 1.015 0.84

LatS_54.81 - LatN_17.6 0.5387 0.1538 3.503 0.004

LatS_35.57 - LatN_38 -1.3977 0.1139 -12.271 < 0.001

LatS_36.42 - LatN_38 -1.6145 0.1382 -11.680 < 0.001

LatS_54.81 - LatN_38 -1.2214 0.1480 -8.251 < 0.001

LatS_36.42 - LatS_35.57 -0.2168 0.1086 -1.996 0.26

LatS_54.81 - LatS_35.57 0.1764 0.1217 1.449 0.59

LatS_54.81 - LatS_36.42 0.3931 0.1444 2.722 0.05

**Supplementary Table S4** Tukey’s contrasts for EPP rates at the different latitudes considered. Each latitude is represented by the term LatN or S, corresponding to the hemisphere, followed by the absolute latitudinal value.

Estimate Std.Err z value Pr(>|z|)

LatN_38 – LatN_17.6 2.1972 0.6872 3.197 0.01

LatS_35.57 – LatN_17.6 2.6201 0.6127 4.276 <0.001

LatS_36.42 – LatN_17.6 1.5041 0.8400 1.791 0.36

LatS_54.81 – LatN_17.6 -0.1054 0.8400 -0.125 0.99

LatS_35.57 – LatN_38 0.4229 0.4864 0.869 0.90

LatS_36.42 – LatN_38 -0.6931 0.7528 -0.921 0.88

LatS_54.81 – LatN_38 -2.3026 0.7528 -3.059 0.02

LatS_36.42 – LatS_35.57 -1.1160 0.6855 -1.628 0.46

LatS_54.81 – LatS_35.57 -2.7254 0.6855 -3.976 <0.001

LatS_54.81 – LatS_36.42 -1.6094 0.8944 -1.799 0.36

**Supplementary Table S5** Length of the breeding season for the populations sampled, and Pearson’s correlations for the variables considered in the table. Length of the breeding season was calculated as the number of days from the appearance of the first egg in the colony until the day the last egg was laid in the colony. EPP: rates of extra-pair paternity, that is percent of nests with at least one extra-pair young in the population.

| **Species name** | **Length of breeding season (in days)** | **EPP rate** | **latitude** |
| --- | --- | --- | --- |
| *T. thalassina* | 38 | 67 | 38 |
| *T. meyeni* | 56 | 13.5 | 54.81 |
| *T. leucorrhoa* | 63 | 61 | 36.42 |
| *T. leucorrhoa* | 71 | 78 | 35.57 |
| *T. albilinea* | 98 | 18 | 17.6 |

| **Variable** | **by Variable** | **Correlation** | **Count** | **Lower 95%** | **Upper 95%** | **Signif Prob** |
| --- | --- | --- | --- | --- | --- | --- |
| latitude | length of breeding season | -0.7185 | 5 | -0.9797 | 0.4473 | 0.1715 |
| EPP rate | length of breeding season | -0.3974 | 5 | -0.9475 | 0.7467 | 0.5077 |
| EPP rate | latitude | -0.0417 | 5 | -0.8912 | 0.8727 | 0.9470 |

**Supplementary Methods** For likelihood calculations in Cervus, we used the following simulation parameters:

WRSW: for 2003-2004 the proportion of fathers sampled in the population for both time periods was 90% (this proportion was taken directly from our field observations of active nests in the area, and the males captured at these nests), 10,000 offspring, 80% relaxed and 95% strict confidence levels, 100% and 97% of loci typed for each time period sampled, respectively, and 1% of loci mistyped, as calculated by the program from our data. With these parameters, we obtained a 99% and 95% assignment rate for each time period under the strict confidence level, respectively.

MANS: for 2003 the proportion of fathers samples in the population was taken from our field observations and was 80%, 10,000 offspring, 95% relaxed and 99% strict confidence levels, 99% of loci typed, and 5% of loci mistyped, as calculated by the program from our data. With these parameters we obtained a 100% assignment rate under the strict confidence level.

VGSW: for 2008-2009 the proportion of fathers sampled in the population for both time periods was 50% (this proportion was taken directly from our field observations of active nests in the area, and the males captured at these nests), 10,000 offspring, 80% relaxed and 95% strict confidence levels, 95% of loci typed for each time period sampled, and 5% of loci mistyped, as calculated by the program from our data. With these parameters, we obtained a 96% assignment rate for both time periods under the strict confidence level, respectively.
